# Patterns of Antibiotic Prescription in Endodontic Therapy in the Republic of Croatia

**DOI:** 10.3390/antibiotics13070645

**Published:** 2024-07-12

**Authors:** Josipa Sović, Sanja Šegović, Božidar Pavelić, Ivona Bago, Ivana Šutej, Ivan Tomašić

**Affiliations:** 1 PhD Postgraduate Study, School of Dental Medicine, University of Zagreb, 10000 Zagreb, Croatia; josipa.sovic@ymail.com; 2 Dental Clinic, Health Center Križevci, 48260 Križevci, Croatia; 3 Department of Endodontics and Restorative Dentistry, School of Dental Medicine, University of Zagreb, 10000 Zagreb, Croatia; pavelic@sfzg.hr (B.P.); bago@sfzg.hr (I.B.); 4 Department of Pharmacology, School of Dental Medicine, University of Zagreb, 10000 Zagreb, Croatia; isutej@gmail.com; 5 School of Innovation, Design and Engineering, Mälardalen University, 721 23 Västerås, Sweden; ivan.tomasic@mdu.se; 6 Department of Communication Systems, Jožef Stefan Institute, 1000 Ljubljana, Slovenia

**Keywords:** antibiotics, endodontic treatment, penicillin with clavulanic acid, bacterial endocarditis

## Abstract

In response to the global trend of decreasing antibiotic usage, this study aimed to evaluate the nature and frequency of antibiotic prescriptions in conjunction with endodontic therapy in Croatia and to assess the attitudes of Croatian dental practitioners towards the endodontic treatment of patients susceptible to bacterial endocarditis. A survey questionnaire was sent to all dental practitioners in Croatia, achieving a response rate of 27%. The most frequently prescribed antibiotic was penicillin with clavulanic acid (63.4%), while standalone penicillin was less prevalent (18.6%). For patients exhibiting penicillin allergies, 90% of respondents indicated clindamycin as their preferred alternative. Antibiotics were mostly prescribed for localized acute apical abscesses without fever, followed by prophylaxis for infectious endocarditis and cellulitis. Only 1.3% of the respondents reported frequent antibiotic prescriptions without accompanying local treatment. While a substantial proportion of surveyed practitioners professed familiarity with the latest guidelines for antibiotic prophylaxis, their choice of antibiotics did not consistently reflect this claim. Most respondents conducted endodontic procedures on patients at risk of bacterial endocarditis. The findings highlight a need for targeted continuing education for dental practitioners in the Republic of Croatia, ensuring their practices align with current guidelines and global trends in antibiotic prescription.

## 1. Introduction

Previous research in Croatia has investigated medication prescription practices in dental offices and their contribution to the country’s national consumption [1,2], especially antibiotic prescriptions [3,4,5]. This is in line with the global trend of reducing antibiotic usage [6] driven by the emergence of resistant strains [7,8]. Previous studies also indicate that approximately 10% of antimicrobial prescriptions in primary care are attributed to dentists [9].

This study aimed to evaluate the prescription patterns of antibiotics by dental practitioners in conjunction with endodontic therapy in the Republic of Croatia. It also aimed to assess whether Croatian dental physicians are amenable to endodontic treatment for patients at risk of bacterial endocarditis and whether they follow the guidelines on the use of antibiotic prophylaxis to prevent bacterial endocarditis. To this end, a questionnaire was designed and sent to all dental practitioners in Croatia.

## 2. Results

**Figure 1:**
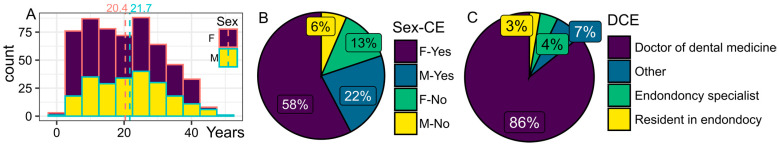
General characteristics of practitioners. (**A**) Years in practice; (**B**) continuing dental education on endodontics in the last 5 years (CE); and (**C**) degree of clinical education (DCE) of the responders (Doctor of Dental Medicine; Specialist in Endodontics; Resident in Endodontics).

A total of 31 responders (3.8%) stated that they do not perform endodontic treatment (female/male: 19/12; mean years in practice: 21). These individuals were excluded from the analysis. Finally, 788 dental practitioners were included in the analysis. Figure 1 presents the general characteristics of the endodontic practitioners who participated in the study, along with the estimates of the population parameters.

We observed that the 788 (92.2%) surveyed dental practitioners performing endodontic procedures in Croatia do not prescribe antibiotics frequently alongside endodontic procedures, according to their subjective evaluation (Table 1).

Table 1 presents data on the frequency of antibiotic prescriptions associated with endodontic therapy, the data on antibiotic usage without any local dental treatment for a particular endodontic issue, the percentage of dental practitioners who are willing to perform endodontic treatment in patients at risk of infective endocarditis, the percentage of dental practitioners who use the rubber dam during endodontic treatment, and the average number of antibiotics prescribed in predefined time intervals.

Regarding the average number of antibiotics prescribed at predefined time intervals (daily/weekly/monthly), the following were the noteworthy observations (Table 1): (i) 122 (15.5%) dental practitioners reported that they prescribed antibiotics at least once a week (which amounted to 488 prescribed antibiotics per month, i.e., 122 × 4); (ii) 336 (42.6%) practitioners prescribed them at least once a month (which amounted to 336 prescribed antibiotics per month); (iii) 14 practitioners (1.8%) prescribed one antibiotic per day (which amounted to 420 prescribed antibiotics per month); (iv) 4 (0.5%) practitioners prescribed at least two antibiotics per day (which amounted to 240 prescribed antibiotics per month); (v) 23 (2.9%) practitioners prescribed at least three antibiotics per week (which amounted to 276 prescribed antibiotics per month). This totals 1760 prescribed antibiotics per month just in the clinical practice of our 788 surveyed dental practitioners (excluding 248 practitioners who administer 1–2 antibiotics over several months).

We found that 34.8% of dental practitioners did not prescribe antibiotics without intervention for susceptible teeth. On the contrary, 47.8% and 16.2% of dental practitioners prescribed antibiotics without local dental treatment very rarely and rarely, respectively (Table 1). Only 1.3% of our respondents reported prescribing antibiotics without local treatment for susceptible teeth. Furthermore, 77.2% of participants were often/almost always/always willing to perform endodontic treatment in patients requiring antibiotic prophylaxis to prevent infectious endocarditis, and 92.5% of participants stated that they were familiar with recent instructions for antibiotic prophylaxis.

Table 2 and Table 3 list the most commonly prescribed antibiotics. Table 4 presents the distribution of antibiotics prescribed (%) for various endodontic diseases. Penicillin in combination with clavulanic acid was the most frequently prescribed antibiotic (63.4%), whereas penicillin alone was less frequently prescribed (18.6%).

**Figure 2:**
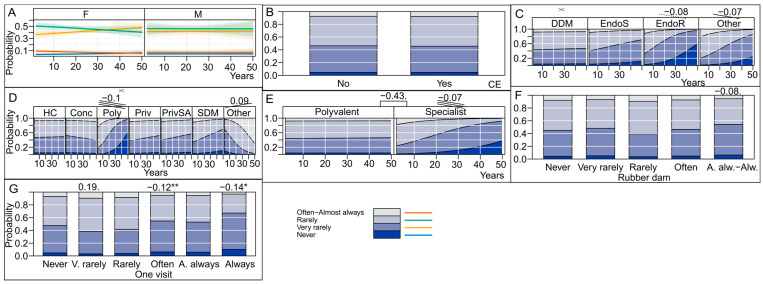
Effect of various factors on antibiotic prescription. (**A**) Effect of sex and years of practice of dental practitioners; (**B**) effect of continuing dental education in endodontics taken by dental practitioners in the last 5 years (CE); (**C**) effect of educational qualifications of the dental practitioner; (**D**) effect of office organization; (**E**) effect of office type; (**F**) effect of rubber dam use; (**G**) effect of “single-visit endodontic treatment”. (Significance codes used are: ** for 0.01, * for 0.05, dot for 0.1. Slopes are designated with corresponding number of stripes whereas a dotted stripe corresponds to a dot, i.e., to 0.1 significance).

**Figure 3:**
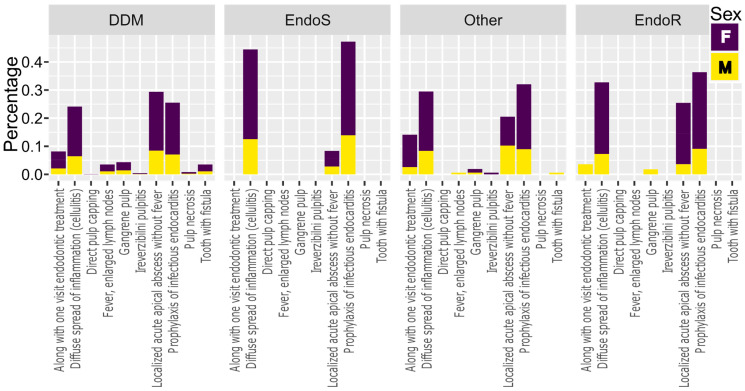
Categorization of dentists prescribing antibiotics for various endodontic etiologies according to sex and educational qualifications. DDM, Doctor of Dental Medicine without specialization; EndoS, Specialist in Endodontics; EndoR, Resident in Endodontics.

**Figure 4:**
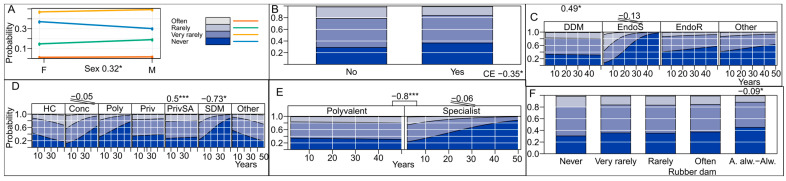
Proportion of dentists prescribing antibiotics without performing treatment on the affected tooth. (**A**) Effect of sex and years of practice of dental practitioners on antibiotic prescription; (**B**) effect of continuing dental education on endodontics taken by dental practitioners in the last 5 years (CE) on antibiotic prescription; (**C**) effect of educational qualifications of the dental practitioner on antibiotic prescription; (**D**) effect of office organization on antibiotic prescription; (**E**) effect of office type on antibiotic prescription; (**F**) effect of rubber dam use on antibiotic prescription. HC, health center; Conc, dental clinic with concession contract; Priv, private clinic; PrivSA, private clinic with a health fund contract; Poly, dental polyclinic; SDM, school of dental medicine; DDM, Doctor of Dental Medicine without specialization; EndoS, Specialist in Endodontics; EndoR, Resident in Endodontics. (Significance codes used are: *** for 0.001, * for 0.05, Slopes are designated with corresponding number of stripes).

**Figure 5:**
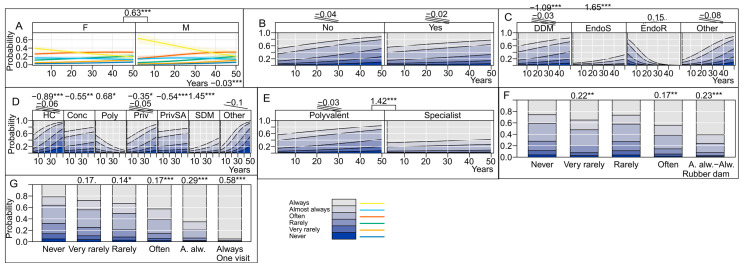
Categorization of dentists willing to perform endodontic treatment in patients at risk of infective endocarditis. (**A**) Effect of sex and years of practice of dental practitioners on antibiotic prescription; (**B**) effect of continuing dental education on endodontics taken by dental practitioners in the last 5 years (CE) on antibiotic prescription; (**C**) effect of educational qualifications of the dental practitioner on antibiotic prescription; (**D**) effect of office organization on antibiotic prescription; (**E**) effect of office type on antibiotic prescription; (**F**) effect of rubber dam use on antibiotic prescription; (**G**) effect of “single-visit endodontic treatment” on antibiotic prescription. HC, health center; Conc, dental clinic with concession contract; Priv, private clinic; PrivSA, private clinic with a health fund contract; Poly, dental polyclinic; SDM, school of dental medicine; DDM, Doctor of Dental Medicine without specialization; EndoS, Specialist in Endodontics; EndoR, Resident in Endodontics. (Significance codes used are: *** for 0.001, ** for 0.01, * for 0.05, dot for 0.1. Slopes are designated with corresponding number of stripes whereas a dotted stripe corresponds to a dot, i.e., to 0.1 significance).

The results of prescribing antibiotics to patients at risk for bacterial endocarditis are presented in Table 5 and Table 6. Answers regarding antibiotic doses for prophylaxis were inconsistent; therefore, these data were not analyzed. Almost all dental practitioners surveyed (92.5%) stated that they were familiar with the recent instructions (updates) on the use of antibiotic prophylaxis in patients at risk for infective endocarditis. The results of regression analysis are presented in Figure 2, Figure 3, Figure 4 and Figure 5.

As seen from Figure 2, no significant effects of “sex”, “years of practice”, “continuing dental education on endodontics taken in the last 5 years”, or “educational qualification” were observed on antibiotic prescription trends. Doctors of dental medicine without specialization showed a significantly different decreasing trend in antibiotic prescription compared to those with other education qualifications; this decreasing trend was significant for endodontic residents (LO = −0.08) and doctors with “other” qualifications (LO = −0.07). Within polyclinics, the rate of prescribing antibiotics dropped significantly over the years −0.1 (for other LO = 0.09); this was significantly different in the specialty clinics (antibiotics were prescribed less [−0.43] and the rate of prescribing antibiotics decreased over the years [−0.07]) compared to non-specialty polyvalent clinics. Figure 2G indicates that those dentists who perform single-visit endodontic treatments prescribe antibiotics less often. For instance, doctors who reported often performing single-visit endodontics are less likely to prescribe antibiotics than doctors who do single visits less often (LO = −0.12).

From the regression analysis in Figure 4, it is noticeable that respondents who confirmed attending endodontic clinical education in the last 5 years, those who worked at the school of dental medicine, and those who use rubber dams almost always or always showed a lower probability of prescribing antibiotics without local intraoral intervention. Years of practice had a positive effect (prescribing fewer antibiotics) on endodontic specialists, practitioners employed in outpatient clinics that provide specialized services, and those employed in outpatient clinics with a concession contract. Doctors of dental medicine without specialization and those working in private clinics with a contract showed a higher probability of prescribing antibiotics without performing an interventional treatment on the tooth.

Our results (Figure 5) indicate that male dental practitioners, endodontic specialists, employees of the school of dental medicine, those who work in outpatient clinics that provide specialized services, those who use rubber dams almost always/always, and those who perform endodontic procedures in one visit often/almost always/always were willing to perform endodontic treatment of teeth in patients at risk of bacterial endocarditis, and these associations were highly significant.

There was a higher statistical probability that dental treatment in patients at risk of bacterial endocarditis will not be performed by those who have been in practice longer, doctors of dental medicine without specialization (with a significant impact of the years of practice), those who work in health centers, in outpatient clinics with a concession contract, and private clinics with a contract. In non-specialist polyvalent services, ambulances, health centers, and private clinics, the influence of years spent in practice was significantly related to the unwillingness of dental practitioners to perform endodontic treatment for patients at risk of bacterial endocarditis. The factor of continuing endodontic education in the last 5 years did not have a significant impact; however, it was observed that the interest in providing such services decreases with the years spent in practice, both for those who have and those who have not undertaken this education. 

## 3. Materials and Methods

This study is part of the dissertation titled “Assessment of procedures in the performance of endodontic therapy in dental offices in the Republic of Croatia”, which was approved by the Ethics Committee of the University of Zagreb.

### 3.1. Questionnaire

Questionnaires were sent to all dental practitioners in Croatia, a group estimated to exceed 3000 in number. The total number of respondents was estimated using the Croatian Institute of Public Health Croatian Health Statistics Yearbook. As of June 2023, 819 responses were collected, representing a response rate of approximately 27% (819/3000). The questionnaire is accessible at the following link: https://forms.gle/nmUeQizSoN2U5SNYA (accessed on 7 July 2024).

All survey participants were asked to provide demographic information including sex, years of practice, educational qualification, type of dental office in terms of the organization (health center, dental clinic with concession contract, private clinic, private clinic with a health fund contract, dental polyclinic, school of dental medicine, emergency dental clinic), type of dental services provided (non-specialty polyvalent/special), continuing dental education on endodontics taken in the last 5 years, and use of rubber dams while performing endodontic treatments. The questions related to antibiotic use are listed below:

Do you prescribe antibiotics in conjunction with endodontic therapy?
  (never/very rarely/rarely/often/almost always/always)

How often do you prescribe antibiotics for endodontic etiology problems?
  (once a day/two or more times a day/once or twice a week/more than twice a week/once or twice a month/once or twice in several months)

What antibiotics are you most likely to prescribe to your patients for endodontic etiology problems?
What antibiotics are you most likely to prescribe to your patients who are allergic to penicillin for endodontic etiology problems?
For which diseases of the endodontic etiology do you most commonly prescribe antibiotics (you can tick more answers)?
  (Reversible pulpitis/Irreversible pulpitis/Pulp necrosis/Gangrene pulp/Localized acute apical abscess without fever/Diffuse spread of inflammation [cellulitis]/Fever, enlarged lymph nodes/Tooth with fistula/Prophylaxis of infectious endocarditis/single-visit endodontic treatment of infected canal)

How often do you prescribe an antibiotic without any local dental intervention on the cause of the endodontic problem?
  (never/very rarely/rarely/often/almost always/always)

Do you accept to perform endodontic treatment in patients who need antibiotic prophylaxis to prevent infectious endocarditis?
  (yes/no)

Are you familiar with the recent instructions (updates) on the use of antibiotic prophylaxis in patients at risk of bacterial endocarditis?
  (yes/no)

What antibiotic and dose do you prescribe for the prophylaxis of bacterial endocarditis in patients who are not allergic to penicillin?
What antibiotic and dose do you prescribe for the prophylaxis of bacterial endocarditis in patients who are allergic to penicillin?

### 3.2. Statistical Methods

The analysis was performed using the R Project for Statistical Computing (ver. 4.3.0) and its associated survey package. The use of a specialized survey package is necessary because the sample size is comparable to the population size, which makes the finite population correction factor non-negligible.

The distribution of practitioners by sex, years of practice, and continuing dental education on endodontics taken in the last 5 years was examined. The Chi-squared test was used to assess the relationship between categorical variables, whereas the analysis of variance (ANOVA) was used to assess the relationship between continuous and categorical variables. Logistic and ordinal regression models were constructed to assess all the factors influencing the variables of interest. All regression models were adjusted for sex and years of practice. For all unordered predictor factors except sex, the sum contrast was used, meaning that the variables were contrasted with their mean. Helmert contrasts were used for the ordered factors.

The finite population correction factor was specified using the Croatian Health Statistics Yearbook published by the Croatian Institute of Public Health, which reports the number of practitioners.

These effects are reported as log odds (LO). Statistical significance was set at *p* < 0.05. The slopes are designated with the corresponding number of stripes, whereas a dotted stripe corresponds to a dot, that is, to 0.1 significance. Bars or bands in the plots stand for 95% confidence intervals. The crosses represent significantly different interactions. All graphs showing the regression analysis results have probabilities on the vertical axis. The tables present the means and standard errors (SEs) of the presented variables.

## 4. Discussion

Our study contributes to the global effort by examining the prescription patterns of antibiotics alongside endodontic therapy in Croatia by dental practitioners.

A recent study [10] emphasized the responsible use of antibiotics. Clinical practitioners were urged to adhere to the following principles: prescribing the most appropriate drug at the correct dose, via the proper route of administration, and for the most appropriate duration. The World Health Organization introduced the AWaRe (Access, Watch, and Reserve) classification of antibiotics in 2021 [11]. In the first group, Access, there are antibiotics that offer the best therapeutic benefit with the lowest potential for resistance. The second group, Watch, includes the agents that are most susceptible to selective resistance. The third group, Reserve, consists of all antibiotics, such as meropenem, which should be used very little, especially in microorganisms that have developed multidrug resistance [12]. Next, the World Health Organization introduced the MIND ME acronym to guide antibiotic use: “M—Microbiology must guide therapy whenever possible; I—Indications should be evidence-based; N—Narrowest spectrum required for effective treatment; D—Dosage appropriate to the site and type of infection; M—Minimize the duration of therapy; E—Ensure monotherapy in most cases” [13]. 

The following alarming findings from the following British and American studies underscore the need for rational antibiotic use: Unnecessary antibiotic therapy is administered in 80% of acute dental disease treatments [14], and 80% of prophylactic antibiotic prescriptions were inappropriate [15]. 

Since 1970, cross-sectional studies have been conducted on antibiotic administration in dentistry, particularly in endodontic clinical practice [7]. Questionnaire surveys have proven to be useful in such studies. In our study, we employed questions related to the types of antibiotics prescribed, the prescribing habits of dentists as determined by their age, sex, educational qualifications, type of clinical practice, and frequency of antibiotic prescriptions for endodontic diseases.

We found that the surveyed dental practitioners performing endodontic procedures in Croatia did not prescribe antibiotics frequently alongside endodontic procedures, according to their subjective evaluation (Table 1). However, regarding the average number of antibiotics prescribed on a daily/weekly/monthly basis, our 788 surveyed endodontic practitioners used to prescribe at least 1760 antibiotics per month. A deeper analysis should assess the distribution of prescribed antibiotics according to the number of patients cared for by the respondents, which was beyond the scope of this study. If we compare the situation in Croatia with that in the UK, we can observe that 40% of dentists in the UK prescribed antibiotics at least three times a week and 15% prescribed antibiotics on a daily basis [6], which indicates a much higher consumption of antibiotics in the UK than that in Croatia. 

Doctors of dental medicine without specialization showed a significantly different decreasing trend in antibiotic prescription compared to those with other education qualifications. The reason for this could be that residents of endodontics acquire knowledge and experience and change their views and habits of prescribing antibiotics over time, as do other specialists, compared to general practitioners.

Within polyclinics, the rate of prescribing antibiotics dropped significantly over the years. Drop with years is significantly different between non-specialty polyvalent services and specialty offices. This may be attributed to the higher degree of knowledge, greater experience, and more time availability for a single endodontic procedure of specialty clinic practitioners than those in non-specialty polyvalent service-provided ambulances. Polyclinics are mainly staffed by specialists and doctors who have been hired for their expertise.

There is some evidence that practitioners who use rubber dams frequently prescribe fewer antibiotics than those who use rubber dams less often (LO = −0.08). There was a significant decrease in antibiotic use among male practitioners who examined more teeth per month. According to our previously reported results [16], almost 73.9% of the endodontic treatments in Croatia are performed by doctors of dental medicine without specialization (17 teeth per month per one doctor) even though it is specialists in endodontics who do most of the treatments (67 teeth per specialist per month corresponding to a total 15% endodontically treated teeth in Croatia per month). For one tooth, the likelihood of antibiotic prescription decreased slightly over time. Dentists who perform single-visit endodontic treatment prescribe the least amount of antibiotics, and this may be because the specialists have adequate equipment and master the techniques of canal instrumentation and disinfection; furthermore, they plan sufficient time to administer high-quality treatment. 

Our results (Table 2) revealed that penicillin in combination with clavulanic acid was the most frequently prescribed antibiotic, whereas penicillin alone was less frequently prescribed, comparable to those of Perić et al., 2015 [3], on the use of antibiotics in Zagreb, capital of Croatia, where penicillin was reportedly prescribed for the treatment of dental diseases in 72.5% of cases, with 57.6% of those involving penicillin combined with clavulanic acid. Mustafa et al. [17] reported a significant increase in the prescription of amoxicillin with clavulanic acid, a broad-spectrum antibiotic, during dental treatment in Kosovo Major Dental Clinics in the years 2015–2019. Šutej et al. [2] noticed a statistically significant increase in co-amoxic unit utilization during the pandemic period for both pandemic years in Croatia. A study [1] reported antibiotics as medicine accounted for 80% of all Croatian dentists’ prescriptions, and amoxicillin with clavulanic acid accounted for 56.4% of all antibiotics prescribed. 

While narrow-spectrum antibiotics are required, broader-spectrum antibiotics may be necessary when needed [17]. It is difficult to defend the choice of a combination of penicillin and clavulanic acid over penicillin alone. In addition, Mustafa et al. [17] expressed concerns about the shift in first-choice antibiotics. Macan et al. [18] found that amoxicillin combined with clavulanic acid was the most effective antibiotic for treating dental infections. In the absence of improvement in two days, supplementation with metronidazole has been recommended in conjunction with local dental intervention for the affected tooth. In another study [19], the antibiotic susceptibility of a panel of bacteria isolated from endodontic infections (98 species) was analyzed, and their antibiotic susceptibilities were as follows: 85% for penicillin V, 91% for amoxicillin, 100% for amoxicillin/clavulanic acid, 96% for clindamycin, and 45% for metronidazole. Other studies [20,21,22,23,24,25] reported that amoxicillin is the antibiotic of choice for treating endodontic infections. A Turkish study [26] reported that the first-choice antibiotic in their study population was ampicillin, while a more recent Turkish study suggested that amoxicillin with clavulanic acid was the antibiotic of choice [27].

Metronidazole was listed as the antibiotic of choice by 1.3% of our respondents, while 2.2% reported penicillin and metronidazole as their first choice. Macan et al. [18] suggested that metronidazole can only be used as an independent therapy in the treatment of acute necrotizing ulcerative gingivitis. For the treatment of odontogenic infections, it is used in combination with other antimicrobial drugs. Palmer [28] stated that metronidazole is an excellent first-line antibiotic for patients allergic to penicillin, those who have recently completed a course of penicillin, or those suspected of having a predominantly anaerobic infection. 

Prescription of several different types of antibiotics (penicillin, metronidazole, and penicillin in combination with clavulanic acid and clindamycin) was reported by few respondents (1.7%), leading to the conclusion that they either did not understand the question or did not follow the instructions for prescribing antibiotics. 

In our investigation, a high percentage of dental practitioners (90%) reported clindamycin as the first-choice antibiotic for patients with penicillin allergies (Table 3), which is comparable to other studies [3,20,21,23,25]; moreover, in our study, prescription of cephalosporins and macrolides was reported by 4 and 4.4% of practitioners, respectively. Erythromycin was reported by Mainjot et al. [22]. Palmer [28] stated that macrolides, such as azithromycin or clarithromycin, should be used as alternatives to penicillin because they are better tolerated than erythromycin. Erythromycin causes nausea, vomiting, and diarrhea in some patients, and many organisms are resistant to erythromycin. Eleazer warned about serious interactions of erythromycin and other macrolides with other drugs [29]. 

Compared with numerous studies [4,17,25,30,31] that highlight incorrect antibiotic prescriptions for conditions lacking clear indications, the results of our study (Table 4) reveal the following trends: Among all prescribed antibiotics, 28.2% was for the localized acute apical abscesses without elevated body temperature (this condition lacks a clear indication for antibiotic use), followed by prophylaxis of infectious endocarditis (26.8%) and diffuse spread of inflammation, i.e., cellulitis (25.2%). However, antibiotic prescriptions for irreversible pulpitis and pulp necrosis were sporadic. Among all prescribed antibiotics, those prescribed for gangrenous pulp represented 4% and those for teeth with sinus tract represented 1.6%. In our pilot study [4] with fewer respondents (*n* = 83), 51% of respondents reported antibiotic administration in cases of localized acute apical abscesses without swelling, 26% in cases of gangrenous pulp, 17% in cases of teeth with fistulas (sinus tract), and 6% in cases of irreversible pulpitis. European Society of Endodontology (ESE) recommendations suggest prescription of systemic antibiotics only for treating acute apical abscesses in medically compromised patients, acute apical abscesses with systemic involvement, progressive infections, and persistent infections; replantation of avulsed permanent teeth; and soft tissue trauma requiring treatment (e.g., sutures, debridement) [6]. Probably, practitioners should consider that antibiotics do not reduce the pain or swelling of the tissue resulting from symptomatic apical pathosis with no evident systemic response nor pain from irreversible pulpitis [14,32]. 

Figure 3 shows the distribution of prescribed antibiotics for different diagnoses and conditions related to endodontic etiology according to the level of clinical education of our respondents. It was observed that endodontic specialists do not administer antibiotics for other than the following conditions: (i) prophylaxis of infectious endocarditis, (ii) diffuse spread of inflammation (cellulitis), and (iii) localized acute apical abscesses without fever. It seems that the conditions of fever and enlarged lymph nodes were not sufficiently clear in the questionnaire; some respondents did not consider these as indicators of systemic infection, as they were listed separately, without the mention of odontogenic infections. The prescription of antibiotics for localized acute apical abscesses without fever, even by endodontic specialists (8%), although not an indication, may be attributed to the concern regarding infection spread in dubious cases. Although antibiotics are considered to be overused, in some cases, it is challenging to determine if the infection might spread and cause life-threatening complications [29]. Altogether, the present results justify the need for additional continuing dental education for more conscientious prescribing of antibiotics among dental practitioners in the Republic of Croatia. 

This situation does not differ from that of other European countries. Mainjot et al. [22] reported the antibiotic prescription patterns in dental practice within Belgium: 63.3% prescribed antibiotics for periapical abscess, while 4.3% for pulpitis. Rodriguez Núñez et al. [20] reported that 40% of active members of the Spanish Endodontic Society prescribed antibiotics for irreversible pulpitis, while 53% of them prescribed antibiotics for necrotic pulp, acute apical periodontitis, and no swelling. Among the members of the Spanish Oral Surgery Society [21], 86% of respondents prescribed antibiotics in cases of irreversible pulpitis, and 71% of them administrated antibiotics in cases of necrotic pulp, acute apical periodontitis, and no swelling. In a Serbian study [25], 31% of the survey respondents prescribed antibiotics in cases of localized acute apical abscesses without systemic involvement. Thus, European and Croatian dental practitioners prescribe antibiotics inappropriately and in excess when treating endodontic diseases. 

Compared to 34.8% of dental practitioners in this study who did not prescribe antibiotics without intervention for susceptible teeth, Mainjot et al. [22] reported that 54.2% of dental practitioners in Belgium administered antibiotics with no local treatment. Because of compromised pulpal blood circulation in pulpal disease, antibiotics cannot reach the pulp and eliminate microorganisms [30,33]; therefore, it is not justified to prescribe an antibiotic without a procedure on a diseased tooth [34]. 

In our study, 77.2% of participants were willing to perform endodontic treatment in patients requiring antibiotic prophylaxis to prevent infectious endocarditis and 92.5% were familiar with recent instructions for antibiotic prophylaxis. This coincides with the values of 76% and 96.7% reported in the pilot study [4], respectively. Considering that 86% of those surveyed in this study were general dental practitioners and only 7% were specialists and residents in endodontics and pedodontics (Figure 1C), we found that a high percentage of non-specialist practitioners performed endodontic procedures in patients at risk of bacterial endocarditis, even though endodontic procedures may be extremely demanding. However, results presented in Table 5 about prescribing antibiotics for the prophylaxis of bacterial endocarditis in patients not allergic to penicillin revealed that only 66% of respondents prescribed the correct therapy for the prevention of bacterial endocarditis. For patients who were allergic to penicillin, most of our respondents prescribed clindamycin (84.3%), followed by macrolides (7.7%) and cephalosporins 4% (Table 6). According to a recent study [35], a single dose of clindamycin may cause complications, including death, from *Clostridioides difficile* infection. Clindamycin may cause more frequent and severe reactions than the other antibiotics used for antibiotic prophylaxis; Willson et al. [36] no longer recommend its use for this purpose. 

We found that male dental practitioners, endodontic specialists, employees of the school of dental medicine, those who work in outpatient clinics that provide specialized services, those who use rubber dams almost, and those who perform endodontic procedures in one visit were significantly more willing to perform endodontic treatment of teeth in patients at risk of bacterial endocarditis. The educational factor of continuing endodontic education in the last 5 years did have a significant impact on the willingness to perform treatment in such patients; however, it was observed that the interest in providing treatment to such patients decreases with the years spent in practice, both for those who have and those who have not undertaken this education. 

## 5. Conclusions

The results of this study show that there is an inappropriate prescription of antibiotics in dental practice in Croatia. The most commonly used antibiotics are penicillin and clavulanic acid, despite the recommendation for the first choice being a narrow-spectrum antibiotic. More than a quarter of prescribed antibiotics were for localized acute apical abscesses without elevated body temperature, which is a condition that lacks a clear indication for antibiotic use. Despite considerations and warnings about complications related to the use of clindamycin, 90% of respondents reported clindamycin as the first-choice antibiotic for patients with penicillin allergies, as well as for patients at risk of bacterial endocarditis who are allergic to penicillin (84.3%). In addition, most respondents stated that they performed endodontic procedures on patients at risk of bacterial endocarditis.

A high percentage of surveyed practitioners stated that they were familiar with the most recent instructions for antibiotic prophylaxis, although this claim was not supported by the appropriate choice of antibiotics. Only 66% of respondents prescribed the correct therapy for the prevention of bacterial endocarditis in patients not allergic to penicillin.

Generally, this paper shows that dentists with higher educational qualifications prescribe antibiotics less often. Still, there is a noticeable need for targeted continuing education for dental practitioners in the Republic of Croatia.

## Figures and Tables

**Table 1:** Evaluation of endodontic practices via the survey questionnaire.

Questions	Dental Practitioners, *n* (%)
**How often do you prescribe antibiotics in conjunction with endodontic therapy?**	
Never	4.6
Very rarely	40.7
Rarely	46.9
Often–Almost always	7.8
**How often do you prescribe an antibiotic without any local dental intervention on the tooth with the endodontic problem?**	
Never	34.8
Very rarely	47.8
Rarely	16.2
Often	1.3
**Do you provide treatment to patients at risk of endocarditis?**	
Never	3.2
Very rarely	6.3
Rarely	13.3
Often	27.3
Almost always	15.5
Always	34.4
**Do you use of rubber dam while performing endodontic treatment?**	
Never	43.3
Very rarely	13.4
Rarely	17.2
Often	11.6
Almost always	6.7
Always	7.9
**What is the frequency of antibiotic prescription along with endodontic therapy on a daily/weekly/monthly basis?**	
One patient per day	1.8
Two or more patients per day	0.5
One or two patients per week	15.5
More than two patients per week	2.9
One or two patients per month	42.6
One or two patients in a few months	31.5
Other	5

**Table 2:** First choice of prescribed antibiotics for endodontic disease.

Types of Antibiotics	Proportion of Dental Practitioners Who Prescribed a Particular Antibiotic (%)
Penicillin	18.6
Penicillin with clavulanic acid (Klavocin, Augmentin)	63.4
Clindamycin (Klindamicin, Klimicin, Dalacin)	6.6
Metronidazole (Medazol)	1.3
Cefalexin	0.1
Penicillin and penicillin with clavulanic acid	5.13
Penicillin and metronidazole	2.2
Several types of antibiotics (penicillin, metronidazole, and penicillin with clavulanic acid and clindamycin)	1.7

**Table 3:** First choice of prescribed antibiotics for treating endodontic disease in patients allergic to penicillin.

Type of Antibiotics	Proportion of Dental Practitioners Who Prescribed a Particular Antibiotic (%)
Clindamycin (Klindamicin, Klimicin, Dalacin)	90
Cephalosporins (Ceporex, Cephalexin)	4
Fluoroquinolones (Ciprofloksacin)	0.1
Macrolides (azitromicin: Sumamed, Azimed, eritromicin)	4.4
Metronidazole (Medazol)	1.4
Wrong answer, wrong therapy (Amoksicilin, Klavocin, Medrol)	0.1

**Table 4:** Distribution of antibiotics prescribed (%) for various endodontic diseases.

Endodontic Diseases	Prescribed Antibiotics (%)
Reversible pulpitis	0.1
Irreversible pulpitis	0.4
Pulp necrosis	0.8
Tooth with fistula	1.6
Fever and enlarged lymph nodes	3.2
Gangrenous pulp	4
Single-visit endodontic treatment of infected tooth	8.2
Diffuse spread of inflammation (cellulitis)	25.2
Prophylaxis of infectious endocarditis	26.8
Localized acute apical abscess without fever	28.2

**Table 5:** Patterns of antibiotic prescriptions for bacterial endocarditis prophylaxis in patients not allergic to penicillin.

Type of Antibiotics	Proportion of Dental Practitioners Who Prescribed a Particular Antibiotic (%)
Penicillin	65.9
Penicillin with clavulanic acid	28.1
Penicillin alone and penicillin with clavulanic acid	1.1
Clindamycin	0.9
Cefalexin	0.2
Metronidazole	0.3
Antibiotics recommended by a specialist cardiologist or primary general practitioner	0.8
There are no such cases in practice	0.3
Wrong and unclear answers–wrong dosage and duration of the therapy	2.3

**Table 6:** Prescribing antibiotics in the prophylaxis of bacterial endocarditis for patients who are allergic to penicillin.

Type of Antibiotics	Dental Practitioners Who Prescribe Antibiotics (%)
Clindamycin	84.3
Macrolides	7.7
Cephalosporins	4
Metronidazole	1.3
Antibiotics recommended by a cardiologist or a primary general practitioner	1.1
Incorrect responses	0.7
No cases of bacterial endocarditis prophylaxis in their practice	0.3

## Data Availability

Data are available on request.
